# Supplementary figures and images for: Pseudoalteromonas is a novel symbiont of marine invertebrates that exhibits broad patterns of phylosymbiosis
Source: bioRxiv. 2025 Aug 22:2025.08.22.671635. Preprint. [Version 1] doi: 10.1101/2025.08.22.671635 (PMC12393529; doi:10.1101/2025.08.22.671635)

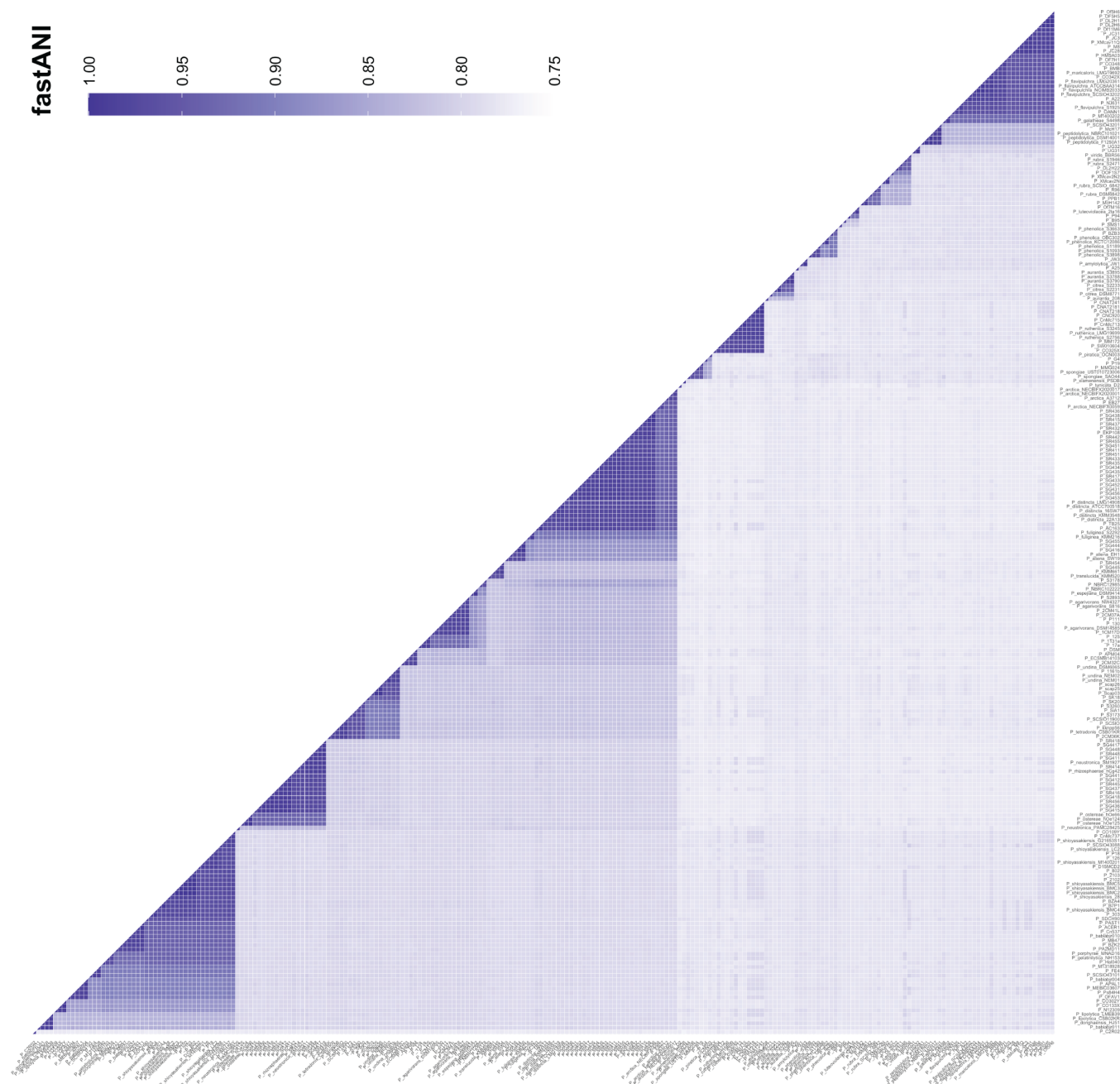

Supplement: Supplement 5 — Extended Data Fig. 1: Average nucleotide identity reveals a higher diversity within the genus Pseudoalteromonas than previously estimated. A higher fastANI score correlates with high genetic similarity between clades. A minimum of 95% fastANI was used to place bacterial isolates in the same phylogroup. [file media-5.pdf]

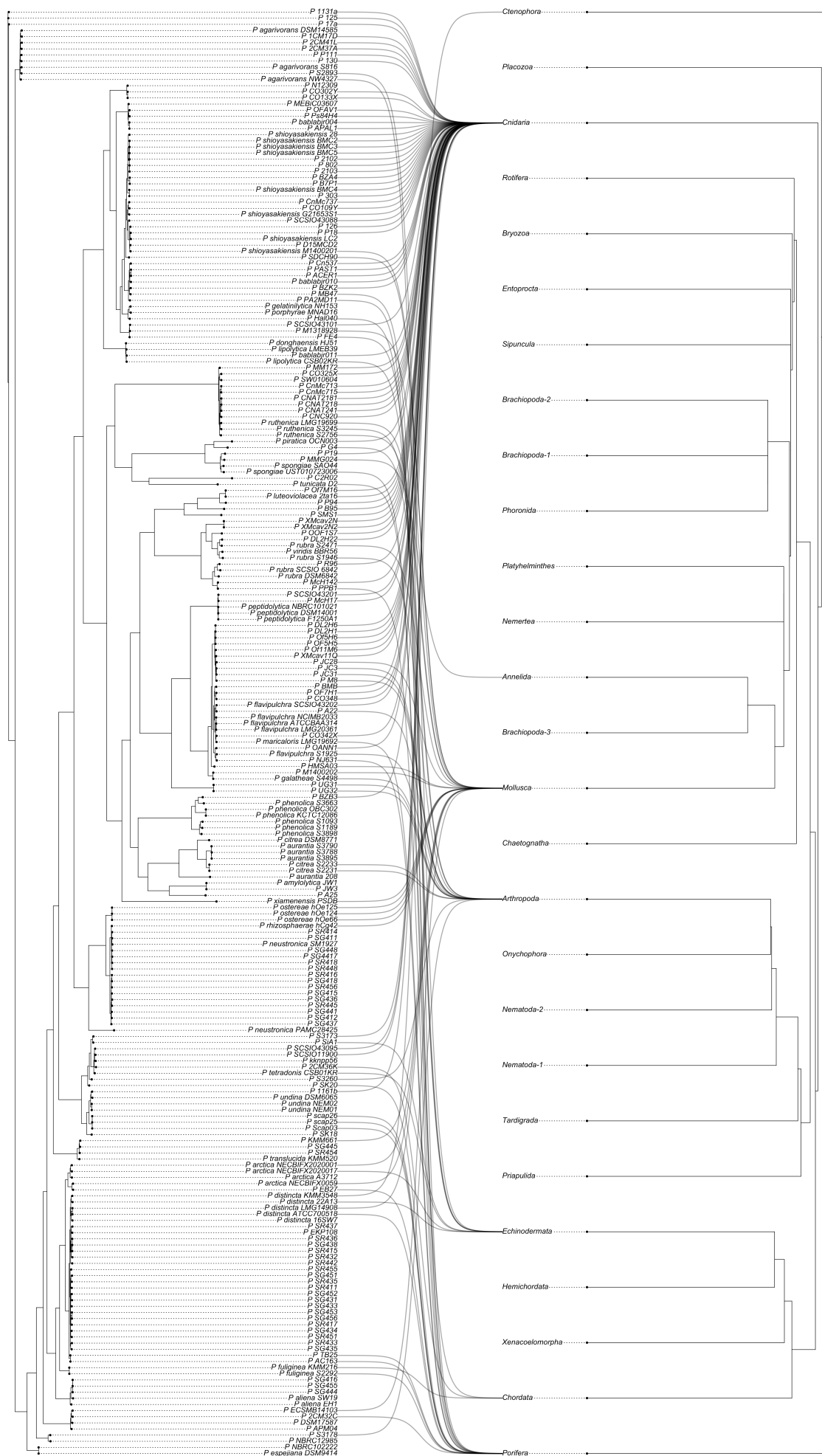

Supplement: Supplement 6 — Extended Data Fig. 2. Additional evidence for cophylogenetic signals between Pseudoalteromonas and marine invertebrates. Phylogenetic trees of Pseudoalteromonas (top) and the hosts (bottom) were used to test for cophylogeny across the Kingdom (Animalia). Topological trees (top) of Pseudoalteromonas were constructed with RAxML (Stamatakis, 2014) using the General Time Reversible (GTR) model with GAMMA-distributed rates across sites and 100 bootstraps. Host cladograms (bottom) were generated via TimeTree (Kumar et al., 2022). Cophylogeny signal was tested using PACo, and phylogenetic congruence was tested using the Generalized Robinson-Foulds metric (Table 1). [file media-6.pdf]

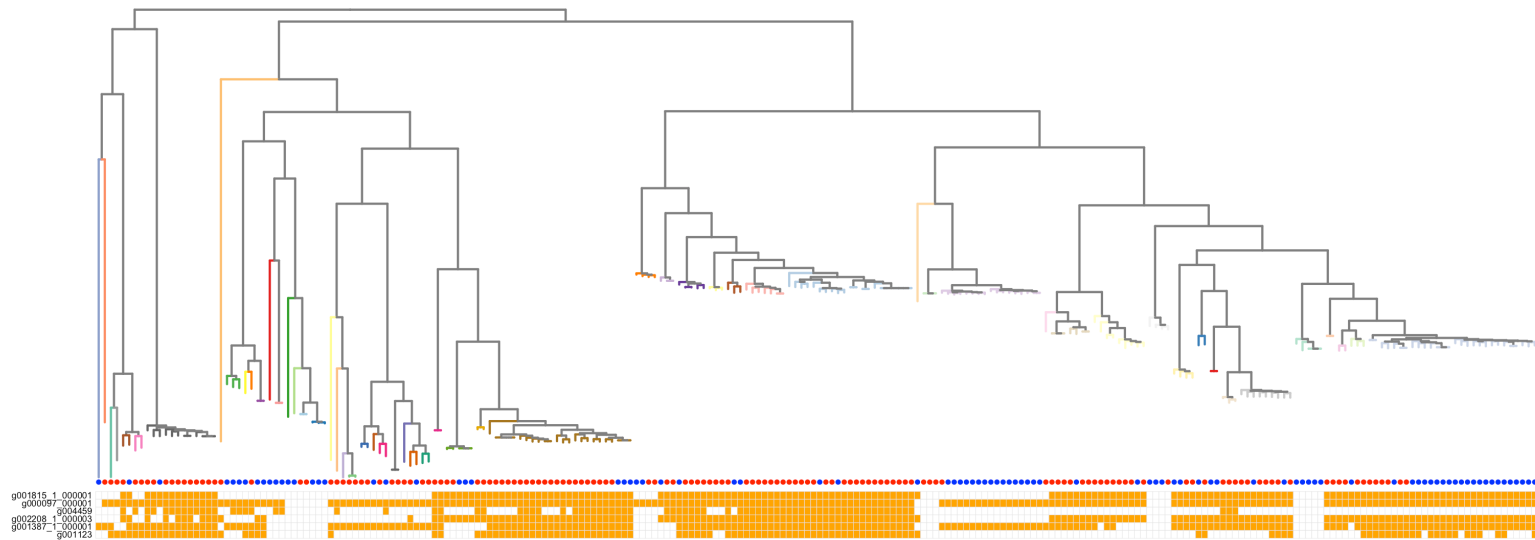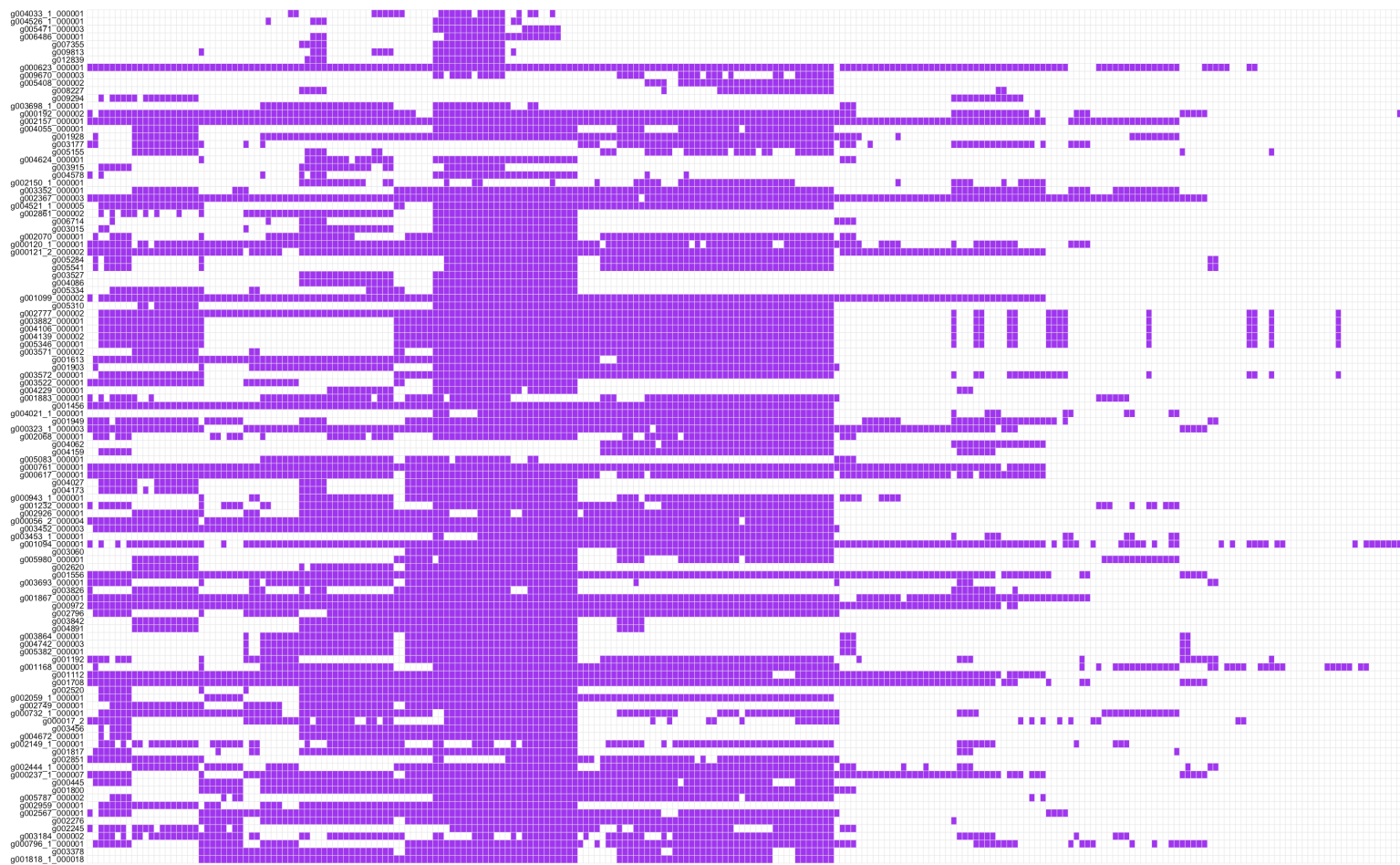

Supplement: Supplement 7 — Extended Data Fig. 3: Host-associated genomes contain distinct sets of genes that enable host-bacterial interactions. A) Midrooted phylogenetic tree of the Pseudoalteromonas pangenome. The tips indicate whether the isolate was a free-living (blue circle) or host-associated (red circle) strain, and the branch color indicates the phylogroup. B) Summary of the accessory genes that were more common in the host-associated pigmented (orange) or host-associated nonpigmented (purple) bacterial genomes. The results of the SCOARY2 panGWAS and a summary of the gene products can be found in Supplementary Table 3. [file media-7.pdf]
